# Enhanced Efficiency and Stability of All‐Inorganic CsPbI_2_Br Perovskite Solar Cells by Organic and Ionic Mixed Passivation

**DOI:** 10.1002/advs.202101367

**Published:** 2021-06-30

**Authors:** Jian He, Jie Su, Zhenhua Lin, Jing Ma, Long Zhou, Siyu Zhang, Shengzhong Liu, Jingjing Chang, Yue Hao

**Affiliations:** ^1^ State Key Discipline Laboratory of Wide Band Gap Semiconductor Technology School of Microelectronics Xidian University 2 South Taibai Road Xi'an 710071 China; ^2^ Advanced Interdisciplinary Research Center for Flexible Electronics Xidian University 2 South Taibai Road Xi'an 710071 China; ^3^ Key Laboratory of Applied Surface and Colloid Chemistry National Ministry of Education Shaanxi Engineering Lab for Advanced Energy Technology School of Materials Science and Engineering Shaanxi Normal University Xi'an 710119 China

**Keywords:** CsBr, CsPbI_2_Br, high efficiency, mixed passivation, PEABr, perovskite solar cells, stability

## Abstract

All‐inorganic perovskites have been intensively investigated as potential optoelectronic materials because of their excellent thermal stability, especially for CsPbI_2_Br. Herein, the authors studied the effects of mixed passivation utilizing organic phenylethylammonium bromide and inorganic ionic cesium bromide (PEABr + CsBr) on the all‐inorganic perovskite (CsPbI_2_Br) solar cells for the first time. The treatment with different passivation mechanisms enhances the perovskite film quality, resulting in uniform surface morphology and compact film with low trap density. Besides, the passivation improves the energy level alignment, which benefits the hole extraction at the perovskite/HTL interface and drives the interface electron separation, suppressing the charge recombination and realizing a high open‐circuit voltage (*V*_oc_). Finally, the device represents a high power conversion efficiency (PCE) of 16.70%, a *V*_oc_ of 1.30 V, and an excellent fill factor (FF) of 0.82. The *V*_oc_ loss and high FF should be among the best values for CsPbI_2_Br based devices. Furthermore, the treated devices exhibit remarkable long‐term stability with only 8% PCE loss after storing in a glove box for more than 1000 h without encapsulation.

## Graphical abstract

Mixed passivation utilizing organic phenylethylammonium bromide and inorganic ionic cesium bromide on the inorganic perovskite CsPbI_2_Br solar cells is studied. The treatment enhances the perovskite film quality, reduces film trap density, improves the energy level alignment, and suppresses the charge recombination. Finally, the device achieves a high power conversion efficiency (PCE) of 16.70%, a open‐circuit voltage (*V*_oc_) of 1.30 V and an excellent fill factor (FF) of 0.82.

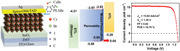

## 1 Introduction

Organic–inorganic hybrid perovskite solar cells (PSCs) have aroused significant interests of many researchers worldwide in the past decade.^[^ ^1^, ^2^, ^3^, ^4^, ^5^, ^6^, ^7^, ^8^, ^9^, ^10^ ^]^ Their inherent superior optoelectronic features have made the power conversion efficiency (PCE) up to the current topmost 25.5% which rivals the commercial monocrystalline silicon solar cells.^[^ ^11^ ^]^ Despite the outstanding performance, organic–inorganic hybrid PSCs still suffer stability issues originated from the organic monovalent cations, such as methylammonium (known as MA^+^ or CH_3_NH_3_^+^) and formamidinium (knowns as FA^+^ or CH(NH_2_)_2_^+^).^[^ ^12^, ^13^, ^14^ ^]^ These organic cations are sensitive to the thermal and moisture circumstances, causing the device deterioration, which impedes the further development.^[^ ^15^, ^16^, ^17^, ^18^ ^]^ To address these issues, the investigations on all‐inorganic halide perovskites CsPb*X*_3_ (*X* = I, Br, Cl) have been carried on owing to the inexistence of the detrimental cations.^[^ ^19^ ^]^

The all‐inorganic CsPb*X*_3_ (*X* = I, Br, Cl), CsPbBr_3_ was studied in the initial stage as its excellent stability.^[^ ^20^ ^]^ Although the CsPbBr_3_ PSC made its debut with a poor PCE of ≈6%, Tang and coworkers recently improved the open‐circuit voltage (*V*_oc_) to an unprecedented level of 1.7 V utilizing a WS_2_/CsPbBr_3_ van der Waals heterostructure to reduce the lattice distortion. As a result, they produced one of the highest PCEs of 10.65% for CsPbBr_3_ PSCs.^[^ ^21^ ^]^ However, the unsatisfied PCE and its wide bandgap (≈2.3 eV) may retard further development. Conversely, CsPbI_3_ has a better appropriate bandgap (≈1.73 eV) for optoelectronic applications.^[^ ^22^ ^]^ Reported by Seok and coworkers lately, the PCE of CsPbI_3_ based PSCs was improved to a new record of 20.37% with highly uniform and pinhole‐minimized thin films processed in the ambient condition by surface engineering.^[^ ^23^ ^]^ Although the narrower bandgap can broaden the absorption of the solar spectrum and then consequently bring the high short‐circuit current density (*J*_sc_) and PCE, the unstable phase transition (the tendency that the film changes from the photoactive black phase to the non‐photoactive yellow phase under the normal circumstances) is an obstacle to achieve good stability.^[^ ^24^ ^]^ Relatively, utilizing mixed anionic CsPbI_2_Br and CsPbIBr_2_ as the study objects is a tradeoff between the optical performance and stability. Among these two materials, CsPbI_2_Br (≈1.93 eV) is more suitable than CsPbIBr_2_ (≈2.08 eV) to win the high efficiency testified by researchers in the past few years.^[^ ^25^, ^26^, ^27^, ^28^ ^]^ Lately, two research groups both realized a PCE of over 17% on the works of CsPbI_2_Br PSCs by adopting different strategies. One substituted A‐site cation with rubidium, and the other passivated the perovskite film through surface engineering. Both approaches not only improved the film quality and reduced the defects, but also achieved good stability.^[^ ^29^, ^30^ ^]^

As mentioned above, the defect passivation is a practical method to improve device performance and stability. Since the surface of the perovskite film is the place where defects often occur, many research works usually filled this kind of defect by employing organic molecules. For example, You and co‐workers deployed the phenylethylammonium iodide (PEAI) as a surface passivation layer on the FA_1−_ *_x_*MA*_x_*PbI_3_ perovskite film to obtain higher efficiency by filling the grain boundaries and suppressing surface defects.^[^ ^31^ ^]^ Then, Ma et al. used a bifunctional dye molecule called YD2‐o‐C8 which could broaden the absorption spectra of the perovskite and reduce the energy loss (*E*_loss_) by interface passivation.^[^ ^32^ ^]^ Liu et al. reported that the nitrogen atom of 1‐ethylpyridinium chloride (EPC) could reduce surface defects by forming chemical bonds with under‐coordinated lead ions and the organic part could simultaneously improve the moisture resistance.^[^ ^33^ ^]^ Besides, the ionic passivation can further reduce the ionic vacancies caused by lattice mismatch^[^ ^34^ ^]^ which can take a role as effective recombination centers.^[^ ^35^ ^]^ Zheng and co‐workers inserted a potassium chloride (KCl) buffer layer between perovskite film and carrier transport layer, observing that the K^+^ and Cl^−^ diffused into the perovskite film, then stopped moving at the bulk defects, and thus enhanced the crystallinity and the device performance.^[^ ^36^ ^]^ He et al. utilized sodium chloride (NaCl) to treat the nickel oxide (NiO*_x_*) film. They found that the Na^+^ fully filled the entire NiO*_x_* film, and it was also detected in the perovskite film at a low level.^[^ ^37^ ^]^ Huang et al. also reported that the Na^+^ diffusing from the substrate could prolong carrier lifetime and reduce trap density, which indicated that the inorganic ions could easily diffuse into the deep part of the perovskite layer where the large organic molecules cannot reach and reduce these defects.^[^ ^38^ ^]^

Herein, we proposed the method that utilizes the mixed organic molecule and inorganic ionic alkali halide (PEABr + CsBr) solution to modify the all‐inorganic perovskite CsPbI_2_Br film through the low‐temperature process for the first time. On one hand, the inorganic ionic alkali halide is effective for ionic defect passivation. Meanwhile, it is thought that the ionic agent could diffuse into the deep perovskite layer where the large organic molecules cannot reach to passivate the defects. On the other hand, the organic molecules can also have a passivation effect by forming low dimensional perovskite or coordinated bonds, as well as molecular interactions on the perovskite film surface or grain boundaries. Merging these two types of mechanisms, the mixed passivation method plays a synergistic role in reducing ionic defects and surface/grain boundary defects all‐around. The mixed passivation could realize the uniform surface morphology and compact perovskite films with low trap density. Moreover, the improved energy level alignment could contribute to the charge extraction and separation enhancement and the recombination suppression at the perovskite/hole transport layer (HTL) interface. As a result, the obtained PSC shows a high PCE of 16.70% with a *V*_oc_ of 1.30 V and a fill factor (FF) of 0.82, and exhibits remarkable long‐term stability with only 8% PCE loss after storing in a glove box for more than 1000 h without encapsulation.

## 2 Results and Discussion

**Figure 1:**
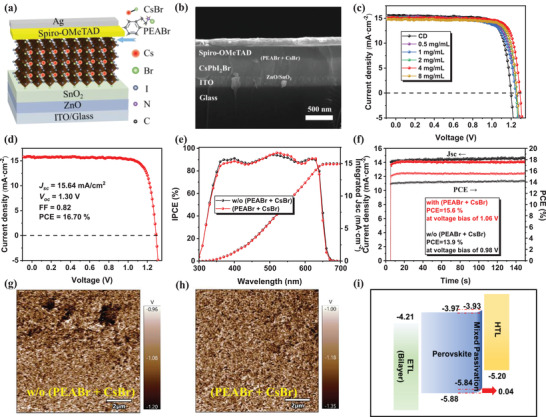
a) Structure and b) cross‐section SEM image of the device. c) *J–V* curves of the PSCs with different concentrations. d) *J*–*V* characteristics of the best device. e) Incident photon‐to‐electron conversion efficiency (IPCE) spectra and f) stabilized power output (SPO) under maximum power‐point conditions of CsPbI_2_Br PSCs without or with the passivation. The KPFM of the perovskite films g) without and h) with the passivation. i) Energy band alignment for each layer in CsPbI_2_Br perovskite solar cells.

The structure of Glass/ITO/ZnO/SnO_2_/CsPbI_2_Br/(PEABr+CsBr)/Spiro‐OMeTAD/Ag is confirmed as the experimental devices, shown as **Figure** **1**a, while the conventional devices are without the (PEABr + CsBr) mixed passivation layer. Figure 1b exhibits the corresponding cross‐section scanning electron microscopy (SEM) image of the completed device. The current density–voltage (*J–V*) measurement was conducted under the ambient condition at room temperature under a light intensity of 100 mW cm^−2^. Figure 1c shows the *J–V* curves of the PSCs treated with passivation layers with different (PEABr + CsBr) concentrations. As the concentration increases, the current density has a slight reduction. It may be attributed to a small increased Br content of the perovskite surface and the increased insulating properties of the passivation layer. Table S1, Supporting Information, gives the corresponding detailed characteristics of the devices. Despite the slightly decreased *J*_sc_, the introduction of the passivation layer has a remarkable benefit on the performance of the PSCs. The device with an optimized 4.0 mg mL^−1^ concentration of (PEABr + CsBr) treatment manifests a much higher average PCE of 15.9% with a high *V*_oc_ of 1.30 V and an excellent FF of 0.81, compared to the control device (CD) which has the average PCE of 14.0%. And the champion device exhibits a PCE of 16.70% with a *J*_sc_ of 15.64 mA cm^−2^, *V*_oc_ of 1.30 V, and FF of 0.82 (Figure 1d). Meanwhile, the individual component passivation was also carried out to give a comparison. Figures S1 and S2, Supporting Information, show the performance of the isolated PEABr and CsBr passivated devices, respectively. Considering that they both have an effect at the same appropriate concentration, the ratio of the two in the mixed solution is used as 1:1. **Table** **1** shows the average photovoltaic performance of CsPbI_2_Br PSCs using different passivation agents. By contrast, the isolated PEABr or CsBr method expresses slight improvement, which means that the mix passivation has a great advantage. And the hysteresis usually caused by the unbalanced charge transfer abilities for electrons and holes is also obviously reduced. As demonstrated in Figure S3 and listed in Table S2, Supporting Information, the hysteresis factor, calculated by the formula of (PCE_Reverse_ − PCE_Forward_)/PCE_Reverse_, decreases from 29.39% to 18.65% (Table S2, Supporting Information), which means that the charge transfer between the perovskite and the HTL after treatment becomes more effective than the conventional one. The incident photo‐to‐current conversion efficiency (IPCE) spectra of the devices with and without mixed passivation are given in Figure 1e. The integrated current densities calculated from the IPCE are 15.0 and 14.9 mA cm^−2^ for the devices without and with passivation, respectively, which are consistent with the *J*_sc_ obtained from the *J–V* measurements within the experimental errors. The stabilized power outputs (SPO) of the treated and untreated devices were measured at their maximum power points for over 150 s, as Figure 1e illustrated. The passivated PSC exhibits a PCE of 15.6%, while the conventional one shows 13.9%. The SPO results match well with the *J–V* results above and reveal the stability under the steady‐state. It also verifies that the device performance has significantly improved after the treatment. To further understand the *V*_oc_ and FF enhancement, the Kelvin probe force microscopy (KPFM) measurement was performed. The information of the surface potential difference is depicted in Figure 1g,h and the corresponding histograms are shown in Figure S4, Supporting Information. The value that draws from the images is the potential difference between the KPFM tip and the perovskite film surface, and is defined as Δ = *W*_F_ (tip) − *W*_F_ (sample). The larger the value of Δ, the smaller its work function will be. As shown in the histograms, the value of Δ for the pristine film is larger than that of the passivated film by 100 mV, which indicates that the perovskite film with the passivation has a lower surface potential and an increased work function. Besides, ultraviolet photoelectron spectroscopy (UPS) measurement, as shown in Figure S5, Supporting Information, also verifies the slight increase in work function, and the Fermi level becomes closer to the valence band, which means an upward band bending occurs and better energy level alignment between perovskite and HTL is achieved, as shown in Figure 1i. The result ensures that the treatment would benefit the charge extraction and separation, reduce the recombination at the perovskite/HTL interface, and finally reach the high *V*_oc_ and FF.

**Table 1:** Average photovoltaic performances of CsPbI_2_Br perovskite solar cells using different passivation agents. The average PCE data were calculated from at least 28 devices

Sample	*J*_sc_ [mA cm^−2^]	*V*_oc_ [V]	FF	PCE [%]
CD	15.4 ± 0.3	1.23 ± 0.02	0.74 ± 0.02	14.0 ± 0.5
PEABr	15.3 ± 0.3	1.27 ± 0.01	0.78 ± 0.01	15.1 ± 0.5
CsBr	15.4 ± 0.4	1.26 ± 0.02	0.76 ± 0.02	14.7 ± 0.6
(PEABr + CsBr)	15.3 ± 0.3	1.30 ± 0.01	0.81 ± 0.01	15.9 ± 0.5

**Figure 2:**
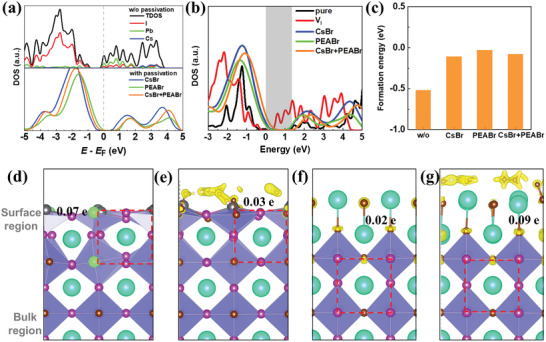
a) Density of states of perovskite surface with *V*_I_. b) Aligned density of states of perovskite surfaces. The gray zone represents the bandgap of pure perovskite. c) Formation energy of *V*_I_ at the perovskite surface. Charge distribution of defective perovskite surface: d) without passivation, with e) PEABr, f) CsBr, and g) (CsBr + PEABr) passivations. The green and yellow regions represent the electron and hole accumulation, respectively. The numbers indicate the amount of charge accumulation. The red dashed box represents the perfect perovskite unit without the disorder.

To unveil the mechanisms and distinction induced by those passivation agents, the micro‐structure and electronic properties of CsPbI_2_Br (001) surface with I‐vacancy (*V*_I_) which generally occurs at the perovskite surface are calculated by density functional theory (DFT). As seen in **Figure** **2**a, since the surface *V*_I_ usually disorders the Pb–I octahedron of perovskite, the evident defect states which can serve as the nonradiative recombination centers are observed in the bandgap of perovskite. Meanwhile, such surface *V*_I_ can bend down the band levels of the perovskite surface (see Figure 2b) since the *V*_I_ induces a number of electrons accumulated at the surface and reduces the energies of Pb–I bonds (see Figure 2d), which results in the mismatch between the band levels of perovskite and HTL. After introducing PEABr passivation, its Br ion can occupy the *V*_I_ defect and then eliminate the structural disorder and defect states of perovskite (see Figure 2a,e), which suggests the reduced nonradiative recombination and enlarged FF for perovskite. Simultaneously, this PEA^+^ cation can supply holes to the perovskite surface (as the hole accumulation at the perovskite surface in Figure 2e) and shift up the band levels of the perovskite surface. It means that the band level mismatch between perovskite and HTL is weakened so that the *V*_oc_ is enlarged after PEABr passivation. Similar characters are also observed for CsBr and mixed (CsBr + PEABr) passivations, as demonstrated in Figure 2f,g. It should be noted that the hole accumulation at the (CsBr + PEABr) passivated perovskite is far more extensive than the isolated PEABr and CsBr passivated perovskites. As a result, the band level of the perovskite surface is more significantly shifted up by the mixed passivation, resulting in a larger *V*_oc_ which is observed in the experiment. In addition, we also calculated the defect formation energies of *V*_I_ which occur at perovskite film surfaces with or without passivation, as depicted in Figure 2c. The result shows that both the isolated CsBr or PEABr passivation and the mixed (CsBr + PEABr) passivation can efficiently suppress the formation of defects existing in the perovskite surface, powerfully contributing to the stability improvement of PSCs.

**Figure 3:**
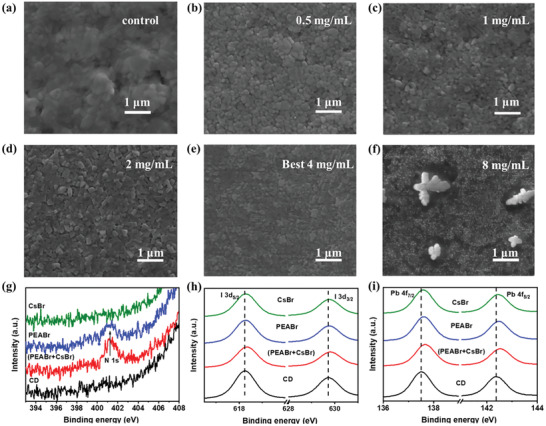
SEM images of the CsPbI_2_Br films a) without and b–f) under different concentrations of the passivating solution. X‐ray photoelectron spectroscopy (XPS) spectra of g) N 1s, h) I 3d, and i) Pb 4f of the CsPbI_2_Br film under different passivation agents.

The surface SEM was measured to study the passivation‐induced morphological changes, as shown in **Figure** **3**a–f. With no passivation, the film exhibits uneven grains with some voids, resulting in poor surface morphology, signifying unsatisfactory device performance. However, as the passivation is applied with the increase of the concentration, the grains become smaller, and the films become smoother and more uniform. Nonetheless, a much higher concentration inversely deteriorates the morphology. As depicted in Figure 3f, superfluous undesired particles severely disrupt the surface topography, leading to rapid performance degradation. The X‐ray photoelectron spectroscopy (XPS) measurement was conducted to further understand the interaction after the passivation. Figure 3g shows that there is an obvious peak of N 1s at 401.23 eV in the spectrum for mixed and isolated PEABr passivated perovskite film, which proves the existence of PEA^+^. For the untreated perovskite film, the peaks of I 3d_3/2_ and I 3d_5/2_ are at around 618.23 and 629.73 eV, respectively. Slightly peak shifts happen after the mixed passivation treatment. Their values increase by 0.10 to 618.33 and 629.83 eV, respectively. However, for the two isolated passivation treatments, the increases of the values are both smaller than the mixed passivation treatment. Specifically, the two isolated passivation treatments both increase the peak values by 0.05 to 618.28 and 627.78 eV. Similar to the situation with iodine, prominent 0.20 eV higher energy shifts can be observed for the peaks of Pb 4f_5/2_ and Pb 4f_7/2_, as for the untreated film, they are at around 137.48 and 142.33 eV, respectively, while for the mixed treated film, they are at 137.68 and 142.53 eV, respectively. The isolated passivation treatments also presented relatively weaker shifts of 0.10 eV for these two peaks. The increased binding energy indicates localized charge transfer occurred between Pb─I octahedral structure and passivated molecules. The result verifies that PEABr and CsBr can play a synergistic effect to realize the potentiation during the mixed passivation. Finally, the vacancy defects are filled and the interaction between ions is enhanced, leading to a more suitable band alignment and thus increases the *V*_oc_ and FF, which is consistent with the calculation result by DFT. The XPS measurements before and after Ar^+^ ion sputter etching were conducted, and the data details are shown in Figure S6, Supporting Information. It shows that the surface of the passivated film has a Br/I ratio of 1.07:1, a Cs/Pb ratio of 1.52:1 and an obvious N content. As the etching time and depth increase, the Br/I and Cs/Pb ratios both decrease to 0.82:1 and 1.41:1, respectively, and the N signal is vanished at the etching time of 50 s. The disappeared N 1s and the modest decrease of Br/I and Cs/Pb ratios indicate that the large radius organic cation (PEABr) mainly remains at the shallow part of the surface and CsBr can reach deeper than the organic cation. After a 600 s etching process, the Br/I ratio keeps a value of about 0.65, which verifies the passivation mechanism we discussed. Figure S7, Supporting Information, describes the UV–Vis spectra of the perovskite films without and with (PEABr + CsBr) treatment. It shows that the light absorption and the bandgap acquired from the absorption onset have almost no change. However, the X‐ray diffraction (XRD) patterns of the CsPbI_2_Br films given in Figure S8, Supporting Information, demonstrate that the diffraction peaks for the treated film are at 14.6°, 20.8°, and 29.5°, corresponding to the (100), (110), and (200) typical crystal planes of the CsPbI_2_Br cubic phase, respectively. The slight intensification of the XRD testifies the quality improvement of the perovskite surface, consistent with the SEM images. Noteworthily, there is a weak peak which appears at the 5.4°, as the asterisk marks, which verifies that the mixed passivation has formed the low‐dimensional perovskite at the surface.

**Figure 4:**
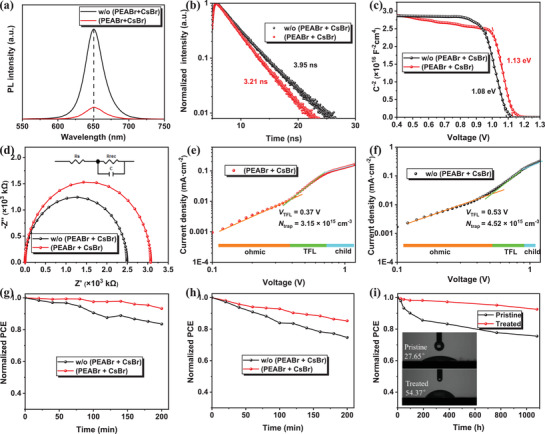
a) PL and b) TR‐PL spectra of the CsPbI_2_Br films. c) *C*–*V* and d) EIS information of the CsPbI_2_Br PSCs. e,f) Dark *J–V* curves for the electron‐only devices with the structure of glass/ITO/ZnO/SnO_2_/CsPbI_2_Br/(with or without (PEABr + CsBr))/PCBM/BCP/Ag. Stability of devices based on different conditions under g) continuous heating at 85 °C, h) continuous light illumination, and i) storing in the N_2_ glove‐box (the insert picture is the contact angle of the perovskite films by utilizing water as the test solvent).

The steady‐state photoluminescence (PL) and time‐resolved photoluminescence (TR‐PL) measurements were further operated to explore the passivation effect on the charge transport process. **Figure** **4**a describes the steady‐state PL results of the devices with and without the treatment. Under normal circumstances, the nonradiative recombination sites will be reduced after passivation, resulting in higher PL intensity and longer TR‐PL time of the perovskite film. However, the quenching at the emission peak is observed. Correspondingly, fitted by the biexponential decay function, the PL lifetime was calculated and is listed in Table S3, Supporting Information, where *A*_1_ and *A*_2_, *τ*1 and *τ*2 refer to the fractions and the lifetimes of fast decay and slow decay, respectively. Clearly, the PL lifetime has reduced from 3.95 (the conventional device) to 3.21 ns (the passivated device), as shown in Figure 4b. This phenomenon can be reasoned over by the upward band bending and enhanced charge transfer. Usually, the steady‐state hole density can balance the density of trapped electrons under illumination. The extra increase in hole density would accelerate the reduction process of trapped electrons and increase the density of available trap sites, resulting in the quenching of the PL and the decrease of the lifetime, which helps the charge transfer at the CsPbI_2_Br/HTL interface.^[^ ^39^, ^40^ ^]^ Figures S9 and S10, Supporting Information, depict the PL and TR‐PL measurements for the films with the HTL. The lower PL intensity and the PL lifetime of 2.49 ns for the structure of pristine perovskite (PVK)/HTL and 1.75 ns for passivated PVK/HTL further verify the more effective charge transfer behavior that happens at the CsPbI_2_Br/HTL interface. Besides, Figure S11, Supporting Information, shows that at the wavelength of around 520 nm, there are small peaks which appear for the isolated PEABr and mixed (PEABr + CsBr) passivated films, while the untreated film shows no peak here, which is also consistent with the formation of low‐dimensional perovskite that draws from the XRD results. Besides, to further verify the enhanced charge transfer and reduced recombination processes of the devices, the transient photovoltage (TPV) and transient photocurrent (TPC) were both conducted and are demonstrated in Figures S12 and S13, Supporting Information. The TPV measurement represents the charge‐recombination time of the devices. It is clear that the passivated device shows a much longer time of 129.1 µs than the untreated device of 78.6 µs, which conveys that the recombination process of the charge carrier has been suppressed, as Figure S12, Supporting Information, depicts. Meanwhile, the TPC measurement is conducted to estimate the charge extraction at the perovskite/HTL interface. Figure S13, Supporting Information, shows that after the passivation, the charge‐extraction time decreases from 1.66 µs for the pristine device to 1.17 µs for the passivated device, indicating the charge extraction process is more effective in the passivated device. Moreover, as depicted in Figure 4c, the capacitance‐voltage (*C*–*V*) curves support carrier extraction and transfer improvement. The built‐in potential (*V*_bi_) extracted from the *C*–*V* curves are 1.13 V for the passivated device and 1.08 V for the untreated one. The increased *V*_bi_ can play a role in the driving force enhancement, giving it a more efficient charge transfer. Furthermore, the electrochemical impedance spectroscopy (EIS) was measured at an applied bias of *V*_oc_ with a frequency range from 1 MHz to 10 Hz and 20 mV AC amplitude under working conditions. The Nyquist plots and relevant fitting data are given in Figure 4d and Table S4, Supporting Information, respectively. A larger recombination resistance (*R*_rec_) for the passivated device indicates increased recombination suppression that happens after the treatment, resulting in the intensification in *V*_oc_ and FF. The SCLC is characterized and shown in Figure 4e,f. In this section, the electron‐only devices were fabricated with the structure of Glass/ITO/ZnO/SnO_2_/CsPbI_2_Br/(with or without (PEABr+CsBr))/PCBM/BCP/Ag and then tested in a dark ambient condition. Concentrating on the intersection of the linear ohmic region and the non‐linear SCLC region, the trap‐filled limit voltage (*V*_TFL_) is obtained. The *V*_TFL_ values are 0.37 and 0.53 V for the perovskite films with and without passivation treatment, respectively. The values of electron trap density can be calculated as 3.15 × 10^15^ and 4.25 × 10^15^ cm^−3^, correspondingly. The reduced trap density confirms the high‐quality film, the efficient charge transfer, and the less *V*_loss_ of the device.

Stability testing is an essential part of assessing device reliability under working conditions, especially when it comes to all‐inorganic perovskite. Its inherent splendid thermal stability, compared to the hybrid perovskite materials, is an important reason for choosing them. The thermal stability testing was carried out by heating the unencapsulated devices at 85 °C in ambient conditions (RH ≈30%) for 200 min incessantly. As described in Figure 4g, the device with (PEABr + CsBr) treatment still maintains more than 92% of the original efficiency, while the untreated one drops to ≈80%. Besides, under room temperature and ≈30% humidity, the illumination stability is obtained by locating the devices without encapsulation under the continuous light illumination of AM 1.5 G, as depicted in Figure 4h. The treated device shows superior stability confronting the continuous light as well. Finally, long‐term stability has taken more than 1000 h with the devices stored in a glove box. After the passivation, the device expressed remarkable stability with only 8% PCE loss, as shown in Figure 4i. The insert picture illustrates the contact angle of the perovskite films by utilizing water as the measure solvent. The doubled contact angle after the passivation indicates that the treated film has a significant enhancement in moisture resistance. Besides, Figure S14, Supporting Information, shows the stability of CsPbI_2_Br perovskite films with and without (PEABr + CsBr) passivation in the air (RH ≈ 40%) without encapsulation. It can be intuitively observed that the film with mixed passivation shows a more outstanding hydrophobicity and stability. In brief, the stability has been dramatically improved due to the passivation, whether it is for light, thermal, or long‐term conditions, which would promote the commercialization of PSCs.

## 3 Conclusion

In summary, we have studied the effects of mixed passivation utilizing (PEABr + CsBr) solution on the all‐inorganic perovskite CsPbI_2_Br PSCs. The results give that this passivation can dramatically improve the morphology of the perovskite films and reduce trap density. The passivation endows a better energy band alignment, which improves the extraction of the hole and the separation of the electron at the perovskite/HTL interface, generating a high *V*_oc_ of 1.30 V. Finally, the passivated PSCs with excellent stability have increased about 17% improvement on the basis of conventional devices to 16.7% in PCE, 1.30 V in *V*_oc_, and 0.82 in FF, respectively. This work provides design insights to achieve more efficient all‐inorganic PSCs with low *V*_loss_.

## 4 Experimental Section

### Materials

Cesium iodide (CsI, 99.998%), lead bromide (PbBr_2_, 99.999%), lead iodide (PbI_2_, 99.999%), phenethylammonium bromide (PEABr, 99.5%), and Spiro‐OMeTAD were purchased from Xi'an Polymer Light Technology Corp. Cesium bromide (CsBr, 99.9%) and SnO_2_ colloid precursor (tin (IV) oxide) were purchased from Alfa‐Aesar. Dimethyl sulfoxide (DMSO, 99.8%), bis‐(trifluoromethane) sulfonimide lithium salt (LiTFSI, 96% purity), 4‐tBP (96% purity), methanol (99.8%), chlorobenzene (99.8%), 2‐methoxyethanol (99.8%), ethanolamine (99.5%), and zinc acetate dihydrate (Zn(CH_3_COO)_2_ · 2H_2_O, 99.0%) were purchased from Sigma‐Aldrich. Tris(2‐(1H‐pyrazol‐1‐yl)‐4‐tert‐butylpyridine)‐cobalt(III)Tris(bis‐(trifluoromethylsulfonyl)imide)) (FK209) was purchased from Dyesol. All the materials were used without purification.

### Film Preparation and Device Fabrication

The indium tin oxide (ITO) glass substrates (10 Ω sq^−1^) were progressively cleaned by ultrasonic treatment with detergent, deionized water, acetone, and alcohol for 30 min per procedure and dried under N_2_ flow. The sol‐gel ZnO precursor solution was made by 100 mg Zn(CH_3_COO)_2_ · 2H_2_O and 28 mg ethanolamine dissolved in 10 mL 2‐methoxyethanol, then stirred for 12 h. The SnO_2_ colloid precursor was diluted into 5% with deionized water. 277 mg PbI_2_, 220 mg PbBr_2_, and 312 mg CsI (PbI_2_:PbBr_2_:CsI = 0.6 mm:0.6 mm:1.2 mm) were dissolved in 1 mL DMSO and then stirred at 70 °C for all night to prepare the CsPbI_2_Br precursor solution. Mixed PEABr and CsBr, with a ratio of equimolar, dissolved in methanol and stirred for all night to make the passivating solution. The Spiro‐OMeTAD solution was formulated with 72.5 mg Spiro‐OMeTAD, 18 µL of LiTFSI solution (520 mg mL^−1^ in acetonitrile), 29 µL of FK209 solution (300 mg mL^−1^ in acetonitrile) and 29 µL of 4‐tBP. After 20 min's UV‐ozone treatment to remove organic residues from ITO substrates for further cleaning, the ZnO precursor solution was coated on the glass to form thin films at 3000 rpm for 45 s and annealed at 150 °C for 30 min. The SnO_2_ films were grown in the same way. The preparing 5% SnO_2_ colloid precursor was coated at 4000 rpm for 30 s on the ZnO films and then annealed at 150 °C for 30 min on a hot plate, too. All the processes above were performed under environmental conditions. Next, the substrates were operated in the N_2_ glove box. Filtering the CsPbI_2_Br solution before utilization, then coating it on the substrates at 500 rpm for 3 s first and then 2500 rpm for 30 s. Following 50 and 150 °C for 2 and 10 min, respectively, the perovskite films were obtained. After that, a passivation layer (mixed PEABr and CsBr solution) was coated on the CsPbI_2_Br film at 3000 rpm for 30 s without annealing. Later, Coating the Spiro‐OMeTAD solution at 1000 rpm for 10 s and 4000 rpm for 45 s, the HTL finished. In the end, 100 nm Ag electrodes were deposited via thermal evaporation. The active area of each device was 0.075 mm^2^.

### Device Characterization

The *J–V* measurements were carried out under the ambient conditions at room temperature utilizing Keithley 2400 under a standard solar simulator with an intensity of 100 mW cm^−2^, calibrating against an NREL certified silicon reference solar cell. Each device has an active area of 0.075 cm^2^. IPCE measurements were conducted by the SCS10‐X150 systems (Zolix instrument. Co. Ltd). Bruker D8 Advance XRD measured XRD measurements. The UV–vis absorption spectra were operated with Perkin–Elmer Lambda 950 spectrophotometer. PL and TR‐PL spectra were obtained via using the Pico Quant Fluotime 300 with a 510 nm picosecond pulsed laser. The morphology characterization of the films was measured by SEM (JSM‐7800F). Ultraviolet photoelectron spectroscopy (UPS) was performed by PHI 5000 VersaProbe III with He I source (21.22 eV) under an applied negative bias of 9.0 V. The XPS was measured by Thermo Scientific K‐Alpha+. Transient photocurrent (TPC) measurement was performed with a system excited by a 532 nm (1000 Hz, 3.2 ns) pulse laser. Transient photovoltage (TPV) measurement was performed with the same system excited by a 405 nm (50 Hz, 20 ms) pulse laser. A digital oscilloscope (Tektronix, D4105) was used to record the photocurrent or photovoltage decay process with a sampling resistor of 50 Ω or 1 MΩ, respectively.

### Simulation Section

All first‐principle calculations were based on DFT as implemented in the Vienna ab initio simulation package (VASP) code with projector augmented wave (PAW) method.^[^ ^41^, ^42^, ^43^, ^44^ ^]^ The Perdew–Burke–Ernzerhof (PBE) functional within the generalized gradient approximation (GGA) was employed to describe the exchange‐correlation interaction.^[^ ^45^ ^]^ The plane‐wave basis cutoff energy was set to be 400 eV. The convergence criteria for the self‐consistent field energy and residual force on each atom were set to be 10^−5^ eV/atom and 0.01 eV Å^−1^. The CsPbI_2_Br surface with PbI termination was modeled by the 2 × 2 × 1 CsPbI_2_Br (001) surface with a seven atomic‐layer. Meanwhile, an I‐atom at the perovskite was removed to form the I‐vacancy. The CsBr passivated perovskite was modeled according to the previous report.^[^ ^46^ ^]^ A vacuum region of more than 15 Å in the *z*‐direction in conjunction with the dipole correction was to avoid the fictitious interaction with its periodic images. The detailed models are illustrated in Figure 2.

## Conflict of Interest

The authors declare no conflict of interest.

## Supporting information

Supporting Information

## Data Availability

Data used to support the findings of this study are included within the article and supplementary information file(s).

## References

[1] M. Liu, M. B. Johnston, H. J. Snaith, Nature 2013, 501, 395. 24025775 10.1038/nature12509

[2] N. Arora, M. I. Dar, A. Hinderhofer, N. Pellet, F. Schreiber, S. M. Zakeeruddin, M. Grätzel, Science 2017, 358, 768. 28971968 10.1126/science.aam5655

[3] D. W. de Quilettes, S. M. Vorpahl, S. D. Stranks, H. Nagaoka, G. E. Eperon, M. E. Ziffer, H. J. Snaith, D. S. Ginger, Science 2015, 348, 683. 25931446 10.1126/science.aaa5333

[4] X. Guo, J. Su, Z. Lin, X. Wang, Q. Wang, Z. Zeng, J. Chang, Y. Hao, iScience 2021, 24, 102276. 33817580 10.1016/j.isci.2021.102276 PMC8005820

[5] J. Di, J. Du, Z. Lin, S. (Frank) Liu, J. Ouyang, J. Chang, InfoMat 2021, 3, 293.

[6] Z. Liu, J. Chang, Z. Lin, L. Zhou, Z. Yang, D. Chen, C. Zhang, S. F. Liu, Y. Hao, Adv. Energy Mater. 2018, 8, 1703432.

[7] B. Zhang, J. Su, X. Guo, L. Zhou, Z. Lin, L. Feng, J. Zhang, J. Chang, Y. Hao, Adv. Sci. 2020, 7, 1903044. 10.1002/advs.201903044 PMC7284208 32537396

[8] J. Di, J. Chang, S. (Frank) Liu, EcoMat 2020, 2, e12036.

[9] L. Zhou, Z. Lin, Z. Ning, T. Li, X. Guo, J. Ma, J. Su, C. Zhang, J. Zhang, S. Liu, J. Chang, Y. Hao, Sol. RRL 2019, 3, 1900293.

[10] J. Ma, Z. Lin, X. Guo, L. Zhou, J. Su, C. Zhang, Z. Yang, J. Chang, S. (Frank) Liu, Y. Hao, Sol. RRL 2019, 3, 1900096.

[11] NREL , Best Research‐Cell Efficiency Chart | Photovoltaic Research | NREL, https://www.nrel.gov/pv/cell‐efficiency.html (accessed: 202011).

[12] A. M. A. Leguy, Y. Hu, M. Campoy‐Quiles, M. I. Alonso, O. J. Weber, P. Azarhoosh, M. Van Schilfgaarde, M. T. Weller, T. Bein, J. Nelson, P. Docampo, P. R. F. Barnes, Chem. Mater. 2015, 27, 3397.

[13] J. A. Christians, P. A. M. Herrera, P. V. Kamat, J. Am. Chem. Soc. 2015, 137, 1530. 25590693 10.1021/ja511132a

[14] T. Leijtens, K. Bush, R. Cheacharoen, R. Beal, A. Bowring, M. D. McGehee, J. Mater. Chem. A 2017, 5, 11483.

[15] J. Huang, S. Tan, P. D. Lund, H. Zhou, Energy Environ. Sci. 2017, 10, 2284.

[16] S. N. Habisreutinger, T. Leijtens, G. E. Eperon, S. D. Stranks, R. J. Nicholas, H. J. Snaith, Nano Lett. 2014, 14, 5561. 25226226 10.1021/nl501982b

[17] M. R. Leyden, M. V. Lee, S. R. Raga, Y. Qi, J. Mater. Chem. A 2015, 3, 16097.

[18] Q. Wang, B. Chen, Y. Liu, Y. Deng, Y. Bai, Q. Dong, J. Huang, Energy Environ. Sci. 2017, 10, 516.

[19] H. Chen, S. Xiang, W. Li, H. Liu, L. Zhu, S. Yang, Sol. RRL 2018, 2, 1700188.

[20] M. Kulbak, D. Cahen, G. Hodes, J. Phys. Chem. Lett. 2015, 6, 2452. 26266718 10.1021/acs.jpclett.5b00968

[21] Q. Zhou, J. Duan, X. Yang, Y. Duan, Q. Tang, Angew. Chem. 2020, 132, 22181.

[22] Y. Wang, T. Zhang, M. Kan, Y. Zhao, J. Am. Chem. Soc. 2018, 140, 12345. 30247030 10.1021/jacs.8b07927

[23] S. M. Yoon, H. Min, J. B. Kim, G. Kim, K. S. Lee, S. I. Seok, Joule 2020, 5, 183.

[24] G. E. Eperon, G. M. Paternò, R. J. Sutton, A. Zampetti, A. A. Haghighirad, F. Cacialli, H. J. Snaith, J. Mater. Chem. A 2015, 3, 19688.

[25] J. He, J. Su, Z. Ning, J. Ma, L. Zhou, Z. Lin, J. Zhang, S. Liu, J. Chang, Y. Hao, ACS Appl. Energy Mater. 2020, 3, 5173.

[26] J. Ma, J. Su, Z. Lin, L. Zhou, J. He, J. Zhang, S. Liu, J. Chang, Y. Hao, Nano Energy 2020, 67, 104241.

[27] L. Yan, Q. Xue, M. Liu, Z. Zhu, J. Tian, Z. Li, Z. Chen, Z. Chen, H. Yan, H.‐L. Yip, Y. Cao, Adv. Mater. 2018, 30, 1802509. 10.1002/adma.201802509 29971864

[28] L. Zhou, X. Guo, Z. Lin, J. Ma, J. Su, Z. Hu, C. Zhang, S. (Frank) Liu, J. Chang, Y. Hao, Nano Energy 2019, 60, 583.

[29] J. V. Patil, S. S. Mali, C. K. Hong, Sol. RRL 2020, 4, 2000164.

[30] J. He, J. Liu, Y. Hou, Y. Wang, S. Yang, H. G. Yang, Nat. Commun. 2020, 11, 4237. 32843644 10.1038/s41467-020-18015-5 PMC7447778

[31] Q. Jiang, Y. Zhao, X. Zhang, X. Yang, Y. Chen, Z. Chu, Q. Ye, X. Li, Z. Yin, J. You, Nat. Photonics 2019, 13, 460.

[32] S. Yang, Z. Guo, L. Gao, F. Yu, C. Zhang, M. Fan, G. Wei, T. Ma, Sol. RRL 2019, 3, 1900230.

[33] X. Wu, L. Zhang, Z. Xu, S. Olthof, X. Ren, Y. Liu, D. Yang, F. Gao, S. Liu, J. Mater. Chem. A 2020, 8, 8313.

[34] H. Si, C. Xu, Y. Ou, G. Zhang, W. Fan, Z. Xiong, A. Kausar, Q. Liao, Z. Zhang, A. Sattar, Z. Kang, Y. Zhang, Nano Energy 2020, 68, 104320.

[35] S. Yun, X. Zhou, J. Even, A. Hagfeldt, Angew. Chem., Int. Ed. 2017, 56, 15806. 10.1002/anie.201702660 28544169

[36] P. Wang, J. Wang, X. Zhang, H. Wang, X. Cui, S. Yuan, H. Lu, L. Tu, Y. Zhan, L. Zheng, J. Mater. Chem. A 2018, 6, 15853.

[37] W. Chen, Y. Zhou, G. Chen, Y. Wu, B. Tu, F. Liu, L. Huang, A. M. C. Ng, A. B. Djurišić, Z. He, Adv. Energy Mater. 2019, 9, 1803872.

[38] C. Bi, X. Zheng, B. Chen, H. Wei, J. Huang, ACS Energy Lett. 2017, 2, 1400.

[39] Q. Jiang, Z. Ni, G. Xu, Y. Lin, P. N. Rudd, R. Xue, Y. Li, Y. Li, Y. Gao, J. Huang, Adv. Mater. 2020, 32, 2001581. 10.1002/adma.202001581 32583905

[40] N. K. Noel, S. N. Habisreutinger, A. Pellaroque, F. Pulvirenti, B. Wenger, F. Zhang, Y. H. Lin, O. G. Reid, J. Leisen, Y. Zhang, S. Barlow, S. R. Marder, A. Kahn, H. J. Snaith, C. B. Arnold, B. P. Rand, Energy Environ. Sci. 2019, 12, 3063.

[41] W. Kohn, L. J. Sham, Phys. Rev. 1965, 140, A1133.

[42] P. Hohenberg, W. Kohn, Phys. Rev. 1964, 136, B864.

[43] G. Kresse, J. Furthmüller, Comput. Mater. Sci. 1996, 6, 15.

[44] P. E. Blochl, Phys. Rev. B 1994, 50, 17953. 10.1103/physrevb.50.17953 9976227

[45] J. P. Perdew, K. Burke, M. Ernzerhof, Phys. Rev. Lett. 1996, 77, 3865. 10062328 10.1103/PhysRevLett.77.3865

[46] X. Meng, Z. Wang, W. Qian, Z. Zhu, T. Zhang, Y. Bai, C. Hu, S. Xiao, Y. Yang, S. Yang, J. Phys. Chem. Lett. 2019, 10, 194. 30596242 10.1021/acs.jpclett.8b03742

