# Supplementary material for: Enhanced Efficiency and Stability of All‐Inorganic CsPbI2Br Perovskite Solar Cells by Organic and Ionic Mixed Passivation
Source: Adv Sci (Weinh). 2021 Jun 30;8(17):2101367. doi: 10.1002/advs.202101367 (PMC8425869; doi:10.1002/advs.202101367)
Supplement: Supplementary file 1 — Supporting Information [file ADVS-8-2101367-s001.pdf]

WILEY-VCH

© 2021 Wiley-VCH GmbH

ADVANCED SCIENCE Open Access

Supporting Information

for *Adv. Sci.*, DOI: 10.1002/advs.202101367

Enhanced Efficiency and Stability of All-inorganic $CsPbI_2Br$ Perovskite Solar Cells by Organic and Ionic Mixed Passivation

Jian He, Jie Su, Zhenhua Lin, Jing Ma, Long Zhou, Siyu Zhang, Shengzhong Liu, Jingjing Chang, and Yue Hao*

Supporting Information

Enhanced Efficiency and Stability of All-inorganic $CsPbI_2Br$ Perovskite Solar Cells by Organic and Ionic Mixed Passivation

Jian He, [a] *Jie Su,* [a,b] *Zhenhua Lin,*[a] *Jing Ma,* [a] *Long Zhou,* [a] *Siyu Zhang,* [a] *Shengzhong Liu, Jingjing Chang,* [a,b]* *Yue Hao* [a,b]

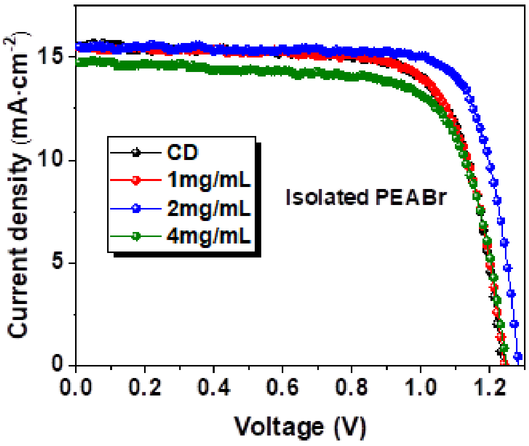

Figure S1. *J* - *V* curves of the isolated PEABr passivated PSCs under different concentrations.

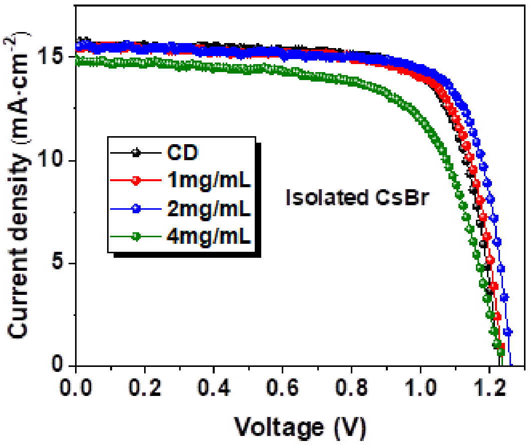

Figure S2. *J* - *V* curves of the isolated CsBr passivated PSCs under different concentrations.

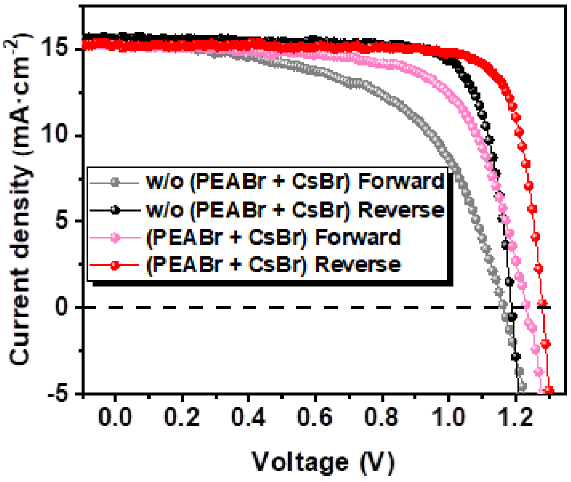

Figure S3. $J - V$ hysteresis characteristics of $CsPbI_2Br$ PSCs without or with the passivation.

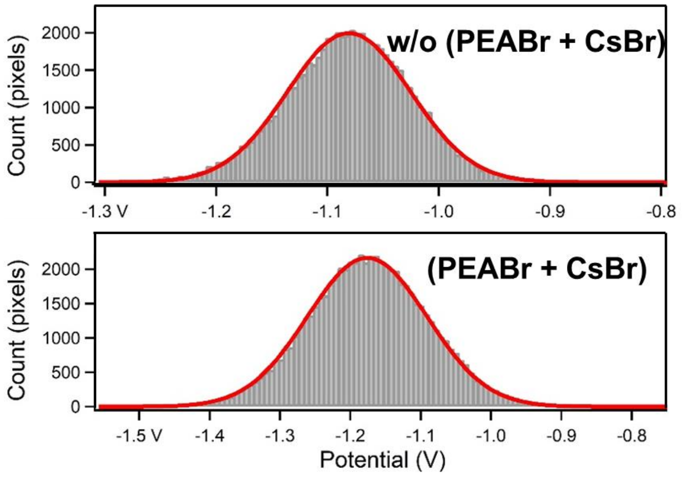

Figure S4. Histogram of the surface potential values measured by KPFM (Kelvin probe force microscopy).

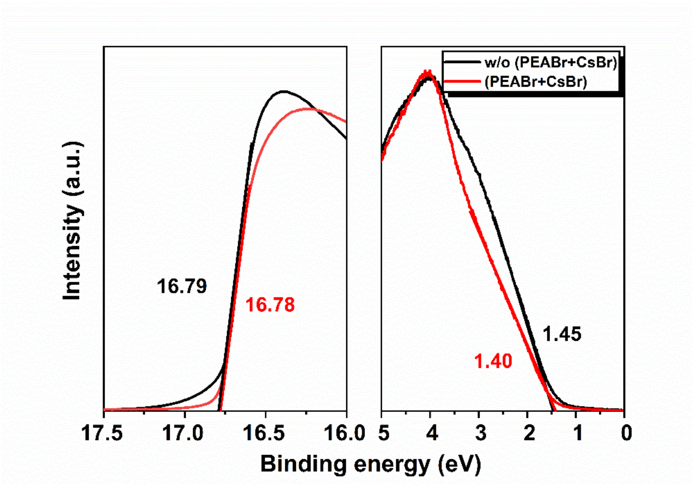

Figure S5. Energy levels of the perovskite film with and without the mixed passivation measured with ultraviolet photoelectron spectroscopy.

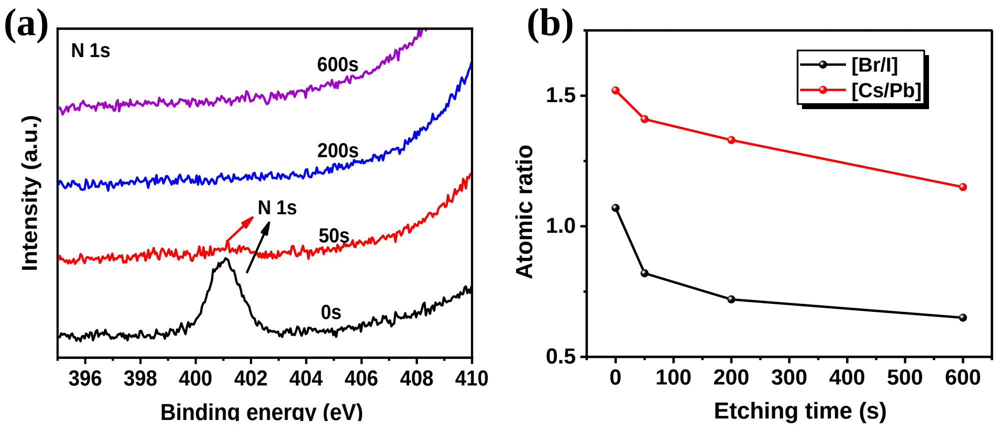

Figure S6. XPS depth-profile analysis of atomic mole ratio changes with etching time.

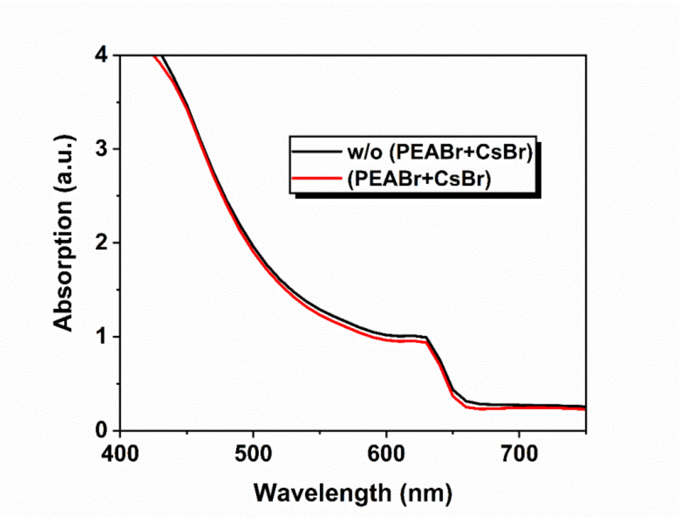

Figure S7. UV-Vis spectra of the perovskite films with and without (PEABr + CsBr) treatment.

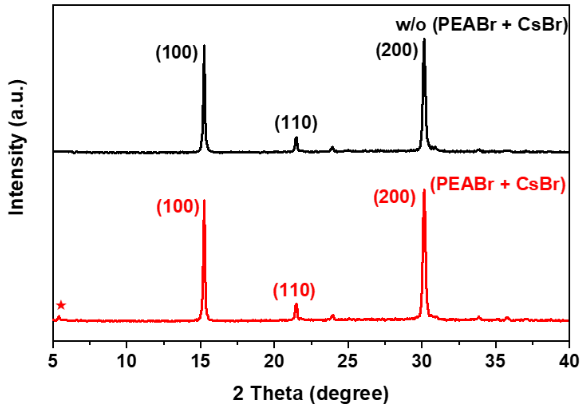

Figure S8. X-ray diffraction (XRD) patterns of the perovskite films with and without (PEABr + CsBr) treatment.

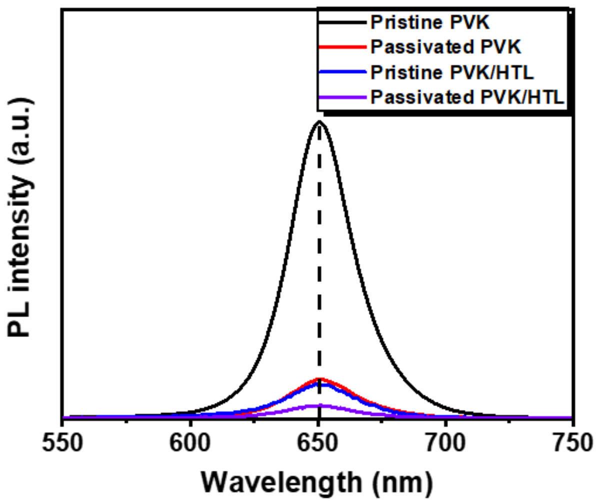

Figure S9. PL spectra of treated and untreated perovskite films with and without HTL, respectively.

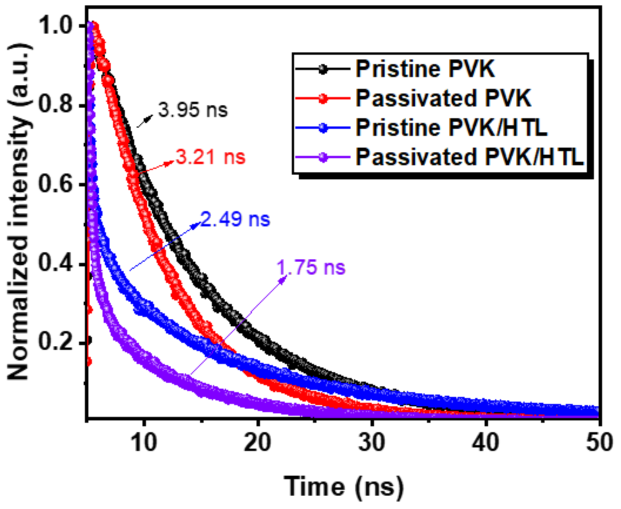

Figure S10. TR-PL spectra of treated and untreated perovskite films with and without HTL, respectively.

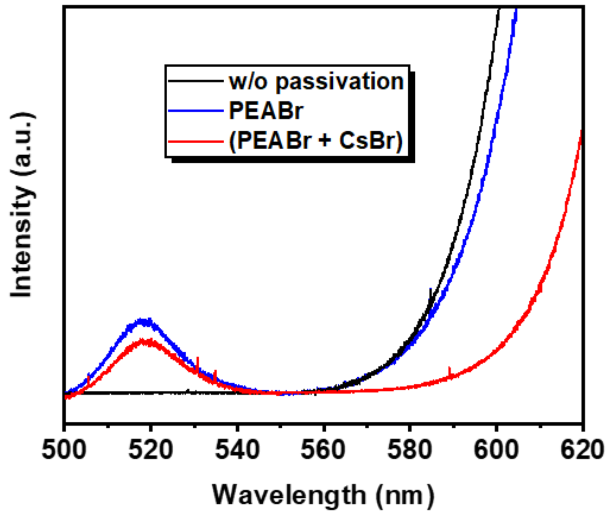

Figure S11. PL spectra of perovskite films with isolated PEABr, mixed (PEABr + CsBr) passivation and without any treatment.

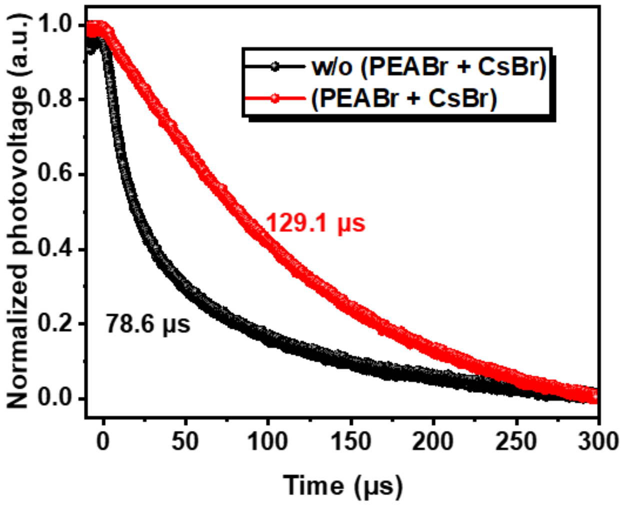

Figure S12. Transient photovoltage (TPV) curve of pristine and passivated $CsPbI_2Br$ perovskite devices

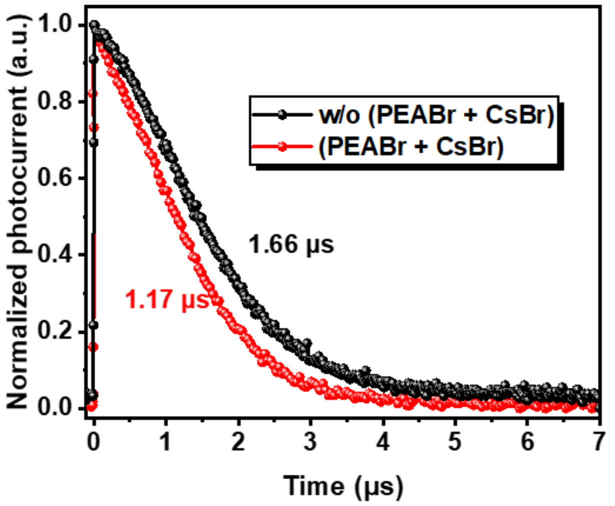

Figure S13. Transient photocurrent (TPC) curve of pristine and passivated $CsPbI_2Br$ perovskite devices

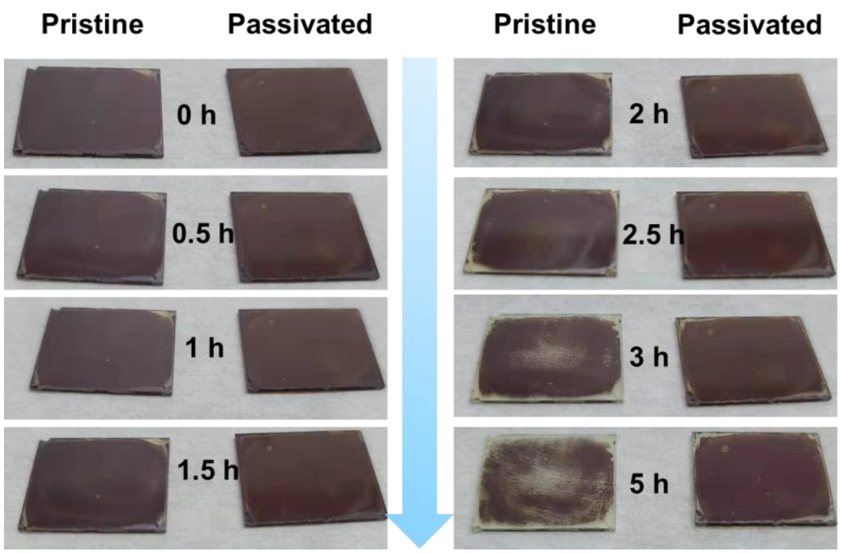

Figure S14. The film stability of the pristine and passivated $CsPbI_2Br$ perovskite films recorded in the air (RH ≈ 40 %) without encapsulation.

Table S1. Average photovoltaic performances of mixed passivated $CsPbI_2Br$ perovskite solar cells.

Concentration (mg/ml)	J_{sc} ($mA \cdot cm^{-2}$)	V_{oc} (V)	FF	PCE (%)
0	15.4	1.22	0.73	13.8
0.5	15.4	1.24	0.76	14.5
1	15.3	1.26	0.78	15.0
2	15.3	1.27	0.78	15.2
4	15.3	1.29	0.81	15.9
8	14.7	1.26	0.79	14.5

Table S2. Summary of the forward and reverse photovoltaic parameters of $CsPbI_2Br$ PSCs.

Sample	V_{oc} (V)	J_{sc} (mA/cm^2)	FF	PCE (%)	HI (%)
CD-R	1.22	15.52	0.73	13.82	29.39
CD-F	1.16	15.49	0.54	9.71	
Mixed-R	1.29	15.25	0.82	16.13	18.65
Mixed-F	1.25	15.20	0.69	13.12	

Table S3. The fitting decay times of perovskite films prepared with different conditions.

	A_1	τ_1 (ns)	A_2	τ_2 (ns)	τ_{avg} (ns)
Pristine PVK	0.27	5.84	0.73	3.24	3.95
Passivated PVK	0.03	7.57	0.97	3.10	3.21
Pristine PVK/HTL	0.99	1.03	0.01	13.50	2.49
Passivated PVK/HTL	0.99	1.11	0.01	11.12	1.75

Table S4. Fitting parameters of the EIS measurement.

	R_s (Ω)	R_{rec} (kΩ)
w/o (PEABr + CsBr)	97.3	2410
(PEABr + CsBr)	189	3071
